# Ellagic acid suppresses the human renal carcinoma cell migration and invasion by targeting the RUNX2/MMP1 expression

**DOI:** 10.7150/ijms.112117

**Published:** 2025-04-22

**Authors:** Po-Yu Huang, Tung-Wei Hung, Yi-Hsien Hsieh, Pei-Jen Wu, Pei-Ni Chen, Chu-Che Lee, Jen-Pi Tsai

**Affiliations:** 1 Division of Nephrology, Department of Internal Medicine, Dalin Tzu Chi Hospital, Buddhist Tzu Chi Medical Foundation, Chiayi, Taiwan.; 2 Institute of Medical Sciences, Tzu Chi University, Hualian, Taiwan.; 3 School of Medicine, Chung Shan Medical University, Taichung, Taiwan.; 4 Division of Nephrology, Department of Medicine, Chung Shan Medical University Hospital, Taichung, Taiwan.; 5 Institute of Medicine, Chung Shan Medical University, Taichung, Taiwan.; 6 Department of Medical Research, Chung Shan Medical University Hospital, Taichung, Taiwan.; 7 Department of Medicine Research, Buddhist Dalin Tzu Chi Hospital, Chiayi, Taiwan.; 8 School of Medicine, Tzu Chi University, Hualian, Taiwan.

**Keywords:** ellagic acid, renal cell carcinoma, MMP1, RUNX2, migration, invasion

## Abstract

Ellagic acid (EA) exerts anti-carcinogenic activity in various types of cancer. Matrix metalloproteinases (MMPs) are critical mediators in the pathogenesis of renal cell carcinoma (RCC) metastasis. Using *in vitro* experiments, this study aims to investigate the mechanisms by which EA inhibits RCC migration and invasion. The findings show that EA treatment inhibited RCC cell migration and invasion without reducing cell viability in normal human kidney cells (HK2 cells) and RCC cells (786-O and ACHN). A human proteinase array showed that EA treatment decreased MMP1 mRNA and protein expression levels in 786-O and ACHN cell lines. MMP1 expression is elevated in RCC tissues and correlates with tumor grade, stage, and overall survival in RCC patients. Our molecular docking model indicates a strong interaction between EA and MMP1. The addition of recombinant human MMP1 (Rh-MMP1) to RCC cells increased their migration and invasion; co-treatment with Rh-MMP1 and EA effectively reversed these effects. EA reduced the expression of the transcription factor RUNX2 in both RCC cell lines and knockdown of RUNX2 significantly decreased the migration and invasion abilities of EA-treated 786-O cells. High expression of RUNX2 in RCC patients is associated with higher tumor grade, stage, and poorer survival and correlates positively with MMP1 expression level. These results suggest that EA suppresses RUNX2 targeting of MMP1 expression, thereby conferring anti-invasive properties on RCC cells.

## Introduction

Renal cell carcinoma (RCC) constitutes the largest proportion of all kidney cancers, with a rising incidence over the past decades 1, 2. The mainstay treatment for advanced RCCs comprises surgical metastasectomy, targeted therapy, and immunotherapy 3, 4. Despite advancements in anticancer therapeutic strategies, the survival rates for RCC remain low 5, 6.

Matrix metalloproteinases (MMPs) exert proteolytic activity in the metabolism of the extracellular matrix (ECM). MMPs contribute to cancer invasion and metastases via proposed mechanisms that include ECM degradation, promotion of the epithelial-to-mesenchymal transition, aberrant angiogenesis, and induction of inflammatory responses 7, 8. Both clear cell and papillary RCCs exhibit much higher expression of MMP1 mRNA than non-tumor tissues. In addition, certain subtypes of tissue inhibitors of matrix metalloproteinases (TIMPs) are downregulated in the RCC microenvironment 9. MMPs have been used as prognostic indicators of advanced RCC. Among patients undergoing immunotherapy for metastatic RCC, higher levels of MMP1 expression were found to correlate with worse progression-free survival 10. MMP2 and MMP9 overexpression in RCC tumors is reported to be associated with a poorer overall patient prognosis 11. In RCC cells, the extent of MMP7 expression was found to correlate positively with the degree of angiogenesis, nuclear grade, cancer stage, and patient survival 12. In summary, various subtypes of MMPs play a critical role in RCC disease progression and related survival.

Ellagic acid (EA), derived from the hydrolysis of ellagitannins found in many plants, is a polyphenol phytochemical involved in a wide variety of physiologic processes 13. EA eliminates myocardial injury, diminishes the risk of cardiac dysrhythmia, retards the progression of neurodegenerative diseases, and preserves liver function 14-16. Furthermore, EA protects against carcinogenesis and cancer invasion through the repression of angiogenesis, induction of apoptotic pathways, reversal of the epithelial-to-mesenchymal transition (EMT), stimulation of DNA repair, and downregulation of pro-inflammatory mediators 17, 18. The molecular mechanisms underlying the anticancer effects of EA have been studied in different types of malignant tumors, including colon cancer, gastric cancer, and hepatocellular carcinoma 19-21. Our previous studies concluded that several naturally occurring compounds can exert anti-metastatic effects on RCC. Corosolic acid (CA) decreases RCC invasion via the regulation of the extracellular signal-regulated kinase (ERK)-MMP2 signaling pathway. Oxyresveratrol decreases MMP1-mediated RCC invasion and migration through the suppression of ERK and protein kinase Cα phosphorylation 22. According to the current literature, the antitumor effects of EA on RCC have not been investigated extensively. This study investigates the molecular mechanisms underlying the suppressive effects of EA on RCC cell migration and invasion.

## Materials and Methods

### Cell lines and culture condition

The RCC cell lines 786-O (clear cell renal cell carcinoma) and ACHN (papillary renal cell carcinoma) were cultured in RPMI-1640 medium. Human normal proximal renal tubular HK2 cells were cultured in DMEM/F12 medium with 10% FBS, 1% penicillin/ streptomycin, and 1% sodium pyruvate. Cells were passaged after reaching 70-80% confluence.

### Cell growth assay

Cell growth rates were assessed using the MTT assay. 786-O, ACHN, and HK2 cells were treated with EA at four concentrations (6.25, 12.5, 25, and 50 μM) for 24 h. After EA treatment, the supernatant medium was removed and ice-cold isopropanol added to dissolve the blue-purple formazan crystals. The OD 570 nm was measured using a spectrophotometer.

### Cell migration and invasion assay

The 786-O and ACHN cells were treated with EA (12.5, 25, and 50 μM) for 24 h. The Boyden chamber assay (non-Matrigel coated) was used to assess cell migration. The lower chamber was filled with 10% FBS-containing medium, followed by placement of an 8-µm cellulose nitrate filter. EA-treated 786-O cells (1 × 10⁴) or ACHN cells (2 × 10⁴) were seeded into the upper chamber and incubated for 16 h (786-O cells) or 18 h (ACHN cells) to assess migration. For the cell invasion assay, the lower chamber set up as for the migration assay. Matrigel (0.5 mg/mL) was added to the upper chamber and incubated for 2 h to allow for gel solidification. The cells were incubated for 20 h (786-O) or 24 h (ACHN). The membranes were removed and fixed with 100% methanol for 30 min and then stained with Giemsa's stain (1:20) for 4 h. The migrated cells were observed under a 400× optical microscope, photographed, and quantified for statistical analysis.

### RNA extraction and qRT-PCR assay

The RCC cells were washed twice with 1 mL of PBS followed by the addition of 1 mL of Trizol reagent and incubation for 2 mins. Chloroform was added, and the mixture was shaken gently up and down for 3 min. After centrifugation for 15 min, the upper aqueous phase (total RNA) was collected and isopropanol added. After centrifugation for 20 min at 4°C, the RNA pellet was air-dried at room temperature. Nuclease-free water was added to dissolve the RNA pellet, and the RNA concentration was assessed using a spectrophotometer. The reverse transcription assay was performed using the GoScript Reverse Transcription Mix (Promega). DEPC-treated water and total RNA were thoroughly mixed, and the RT reaction was conducted under the following conditions: 25°C for 5 min, 42°C for 60 min, and 70°C for 15 min. PCR assays were performed using nuclease-free water, the forward and reverse primers, GoTaq qPCR Master Mix, and cDNA. The PCR tubes were then placed in the StepOnePlus real-time PCR machine and processed using the built-in SYBR-GREEN system settings. The primers of RUNX2 and MMP1 as list: MMP1: F-5'-CTTGCTCATGCTTTTCGACC-3', R-5'-TCCGGGTAGAAGGGATTTGTG-3'; RUNX2: F-5'-CCGGAATGCCTCTGCTGTTATGA, R-5'-ACTGAGGCGGTCAGAGAACAAACT-3'; GAPDH: F-5'-CATCATCCCTGCCTCTACTG-3', R-5'-GCCTGCTTCACCACCTTC-3'.

### Protein extraction and western blot analysis

RCC cells were treated with three different concentrations of EA (12.5, 25, and 50 μM) for 24 h, followed by incubation in NETN protein lysis buffer for 30 minutes. The cells were lysed using ultrasonic homogenization and centrifuged at 13,000 rpm for 30 minutes. The supernatant, containing the total protein fraction, was collected. Proteins were separated via 8-10% SDS-PAGE at 100 V for 1 h and then transferred onto a PVDF membrane in transfer buffer at 100 V for 1 h. The membrane was blocked with 5% nonfat milk for 1 h and then incubated with the MMP1 (SC-21731; dilution 1:1000, Santa Cruz Biotechnology), RUNX2 (#12556, dilution 1:1000, Cell Signaling Technology, Inc) and GAPDH (60004-1-Ig, dilution 1:1000, Proteintech Group, Inc) at 4°C overnight. The secondary antibody was then added and incubated at room temperature for 1 h, followed by three washes with TBST. Protein bands were visualized using a chemiluminescent substrate (ECL) and quantified using the Cytiva ImageQuant 800 system.

### Clinical database for human RCC tissues

The relationship between MMP-1 and RUNX2 gene expression in renal cell carcinoma (tumor) and normal kidney tissues (normal) was analyzed using data in the TIMER2.0 database (http://timer.cistrome.org/). Data regarding tumor grade, tumor stage, and overall survival of patients with low or high expression of MMP-1 and RUNX2 were taken from the TISIDB database (http://cis.hku.hk/TISIDB/index.php).

### Prediction of MMP1 binding energy in EA treatment using a molecular docking model

MMP1 structure information was imported into ChemBio3D to generate and optimize three-dimensional models, which were then converted to PDB format. The relevant crystal structures of MMP1 were obtained from the PDB database and used for molecular docking analysis. The binding energy between EA and MMP1 was calculated using AutoDock Vina software. The most likely binding conformation was identified and visualized using PyMOL 1.8.

### Statistical analysis

Statistical analysis was performed using SPSS 18.0. Student's t-test was used to determine the significance of differences between the two groups. One-way analysis of variance (ANOVA) was used to analyze data across different groups. Spearman correlation coefficients were used to determine correlations between variables. Statistical significance was set at P < 0.05 or P < 0.01.

## Results

### Effect of EA treatment on cell growth, migration and invasion of human RCC cells

**Figure 1:**
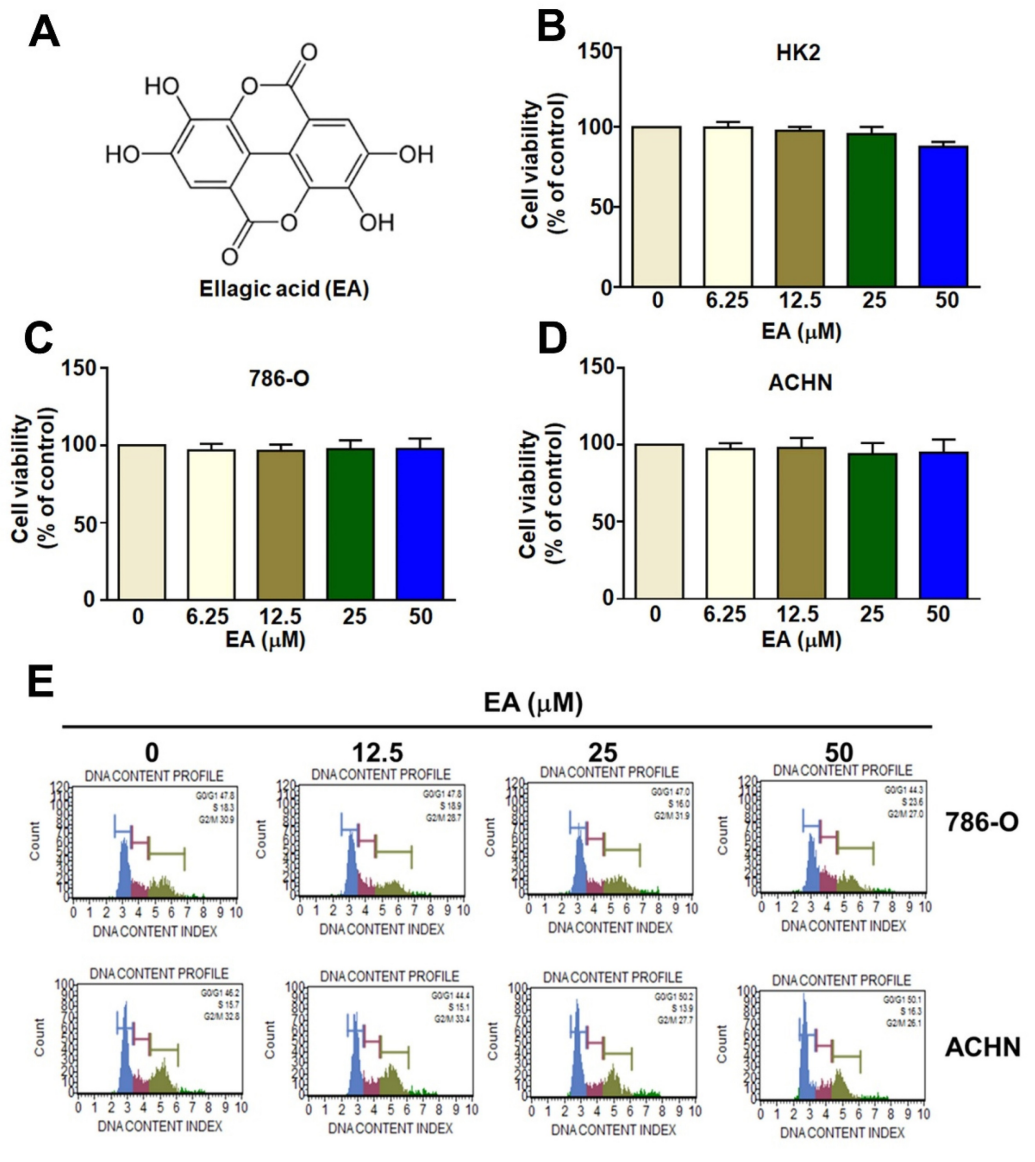
The inhibitory effect of EA on normal renal cells and RCC cell lines. The molecular structure of EA (EA) is shown in (A). Different concentrations of EA were administered during incubation of HK-2 cells (B), 786-O cells (C), and ACHN cells (D) for up to 24 h. The viability of the three cell lines was assessed using the MTT assay. (E) Flow cytometry was used to determine the cell cycle phase of EA-treated RCC cells.

**Figure 2:**
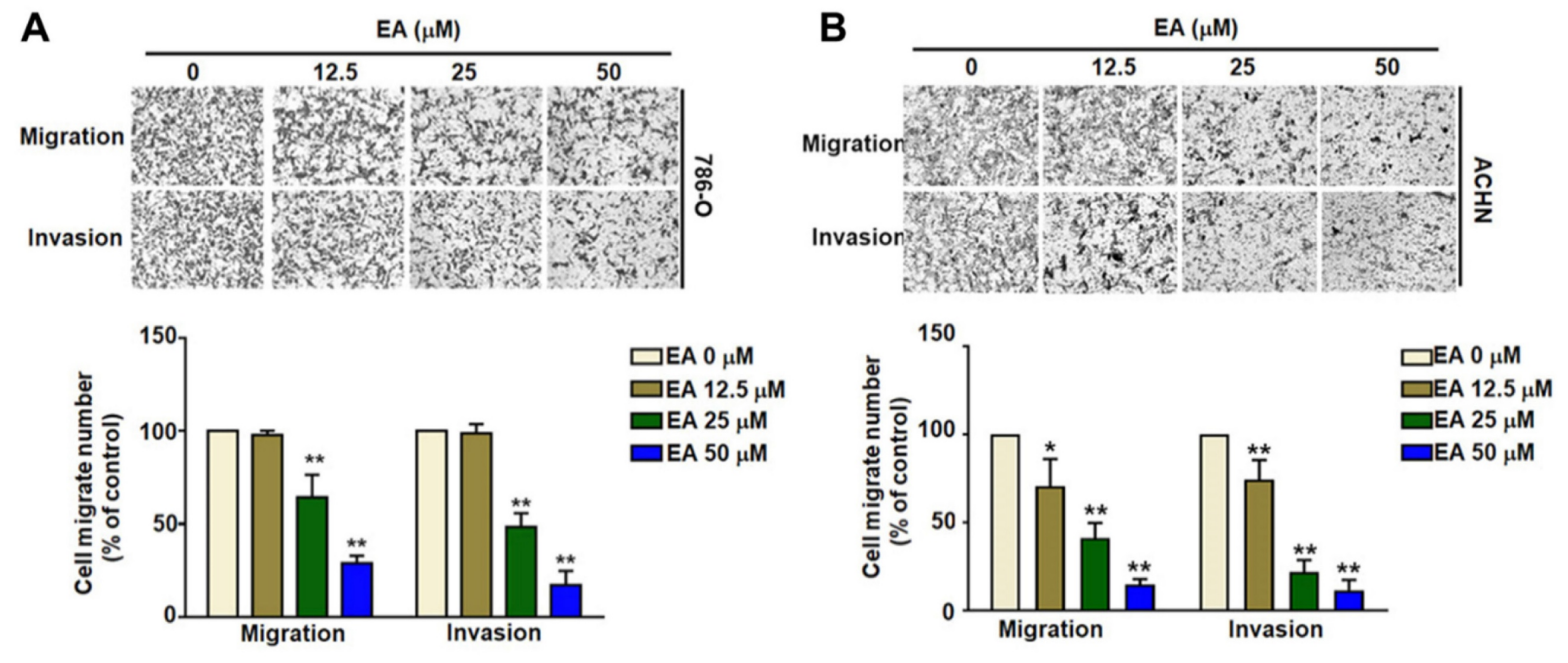
The inhibitory effect of EA on the migration and invasion of human RCC cells. EA treatment inhibited the migration and invasion of the renal cell carcinoma cell lines 786-O (A) and ACHN (B). The histology findings and corresponding histograms of the relative proportions of migrating cells are shown. ** p < 0.01 compared to untreated cells.

The structure of ellagic acid (EA) is shown in Figure 1A. MTT assays comparing the viability of human HK2, 786-O, and ACHN cell lines showed that EA treatment at concentrations up to 50 µM did not significantly reduce the viability of either normal human renal tubular HK2 cells or RCC cells (Figures 1B-1D). Additionally, EA had no effect on the cell cycle in either of the RCC cell lines (Figures 1E). Thus, EA did not exhibit cytotoxicity in normal kidney cells or kidney cancer cells. Treatment of 786-O and ACHN cells with 25 and 50 µM EA resulted in a significant decrease in cell migration (Figure 2A, 2B). Similar results with invasion in EA-treated with 786-O and ACHN cells. These results showed that EA exerts anti-migration and anti-invasion activity on RCC cells.

### EA treatment decreased MMP1 protein expression

**Figure 3:**
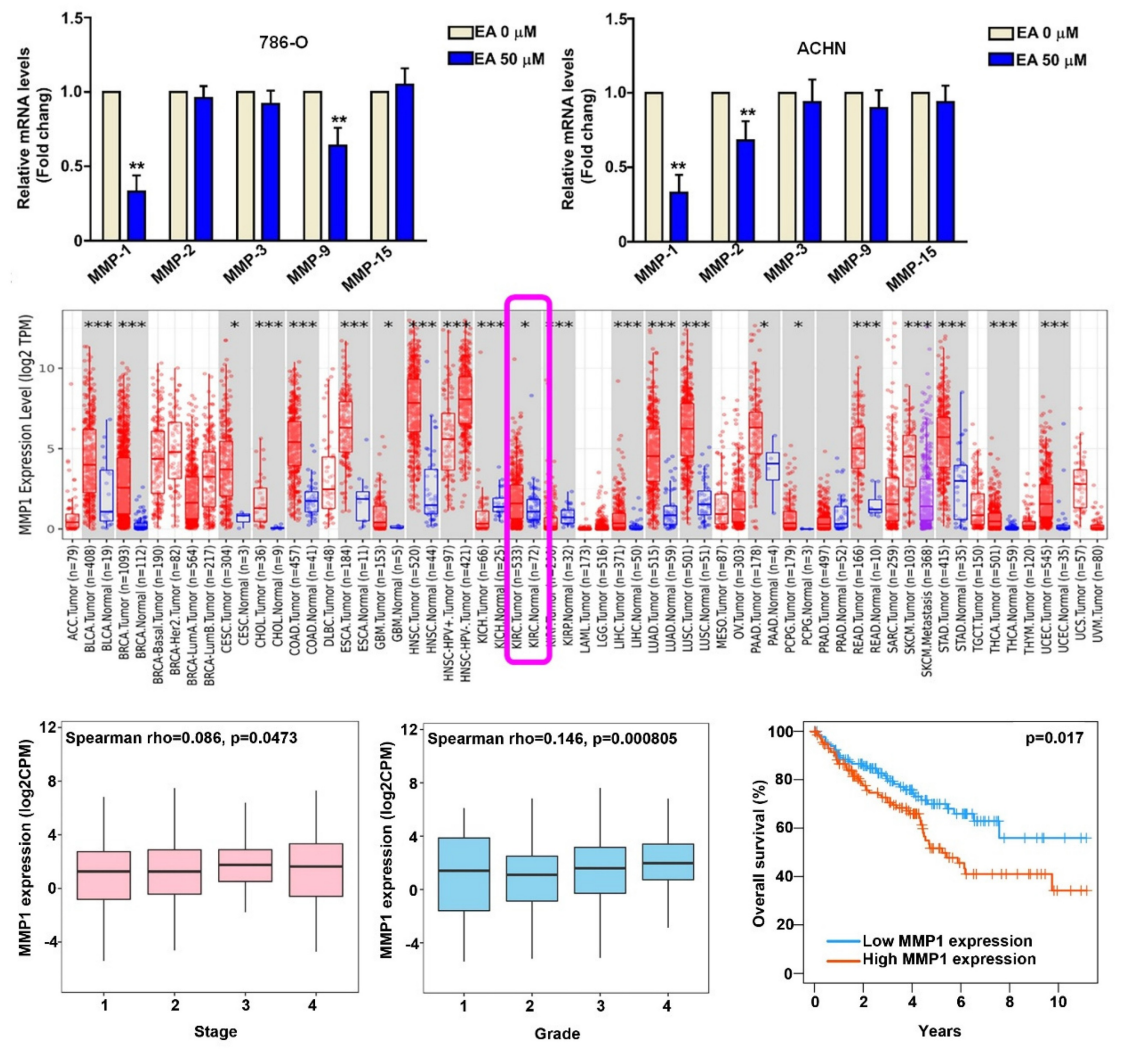
MMP1 expression in EA treated-RCC cells and clinical significance of MMP1 in RCC tissues. (A) Effect of 50-µM EA treatment on MMP expression (MMP1, MMP2, MMP3, MMP9, MMP15) in 786-O and ACHN cells. ** p < 0.01 compared to untreated cells. (B) Analysis of Timer2.0 data comparing MMP1 expression between normal tissues and renal cell carcinoma (RCC) cells. (C) Comparison of tumor stage, tumor grade, and 10-year overall survival rates between high and low MMP1 gene expression levels in RCC tumor tissues.

The effect of EA treatment on the mRNA expression of MMPs (MMP1, MMP2, MMP3, MMP9, MMP15) was assessed using the RT-qPCR assay. Based on the results of cell migration and invasion assays, we used 50 µM EA in further experiments. EA treatment of 786-O and ACHN cells resulted in a significant decrease in mRNA levels of MMP1 compared to untreated cells (Figures 3A). To investigate the pathogenic role of MMP1 in RCC tissues, we analyzed data in the TIMER2.0 database. We found that MMP1 expression was significantly higher in RCC tissues than in normal kidney tissues (Figure 3B) and that higher MMP1 expression was associated with a higher tumor stage (P = 0.0473) (Figure 3C) and tumor grade (P = 0.0008) (Figure 3D) and poorer overall 10-year survival (P = 0.017) (Figure 3E). These findings suggest that MMP1 is a key target of EA and may serve as a prognostic factor for RCC.

### The role of MMP1 in EA-induced decreases in RCC cell migration and invasion

**Figure 4:**
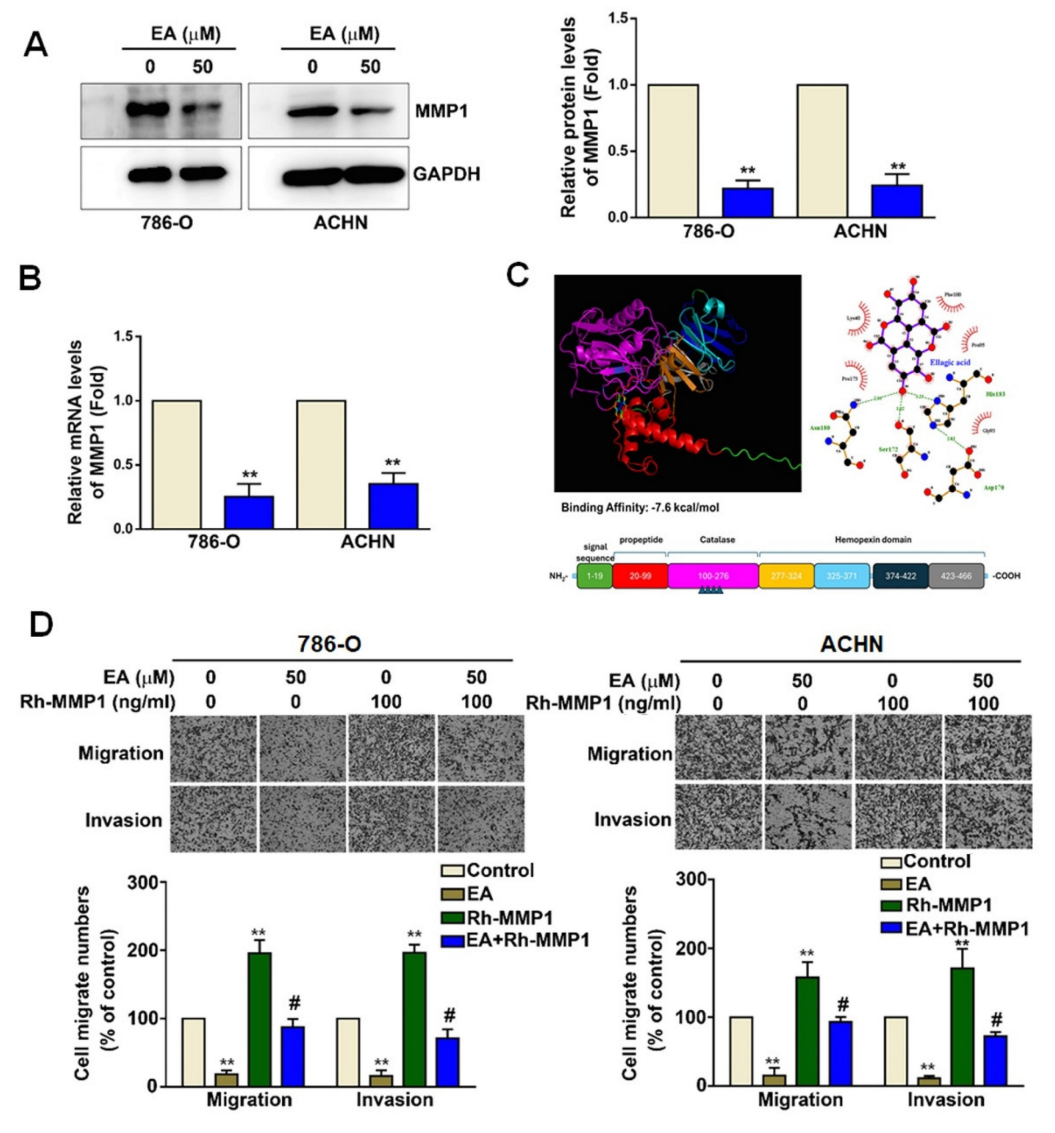
The functional role of MMP1 in EA-treated RCC cells. (A) MMP1 protein expression levels in renal cell carcinoma cell lines with or without EA treatment as assessed by western blot analysis. (B) Relative MMP1 mRNA expression levels as assessed by quantitative reverse transcription polymerase chain reaction (qRT-PCR) assay. (C) Molecular docking of EA and MMP1 protein. (D) EA and recombinant human MMP1 (Rh-MMP1) co-treatment abolished Rh-MMP1-dependent cell migration and invasion in both 786-O and ACHN cells. The histology findings and corresponding histograms of the relative proportions of migrating cells are shown. ** p < 0.01 compared to untreated cells; # p < 0.01 compared to EA-treated cells.

EA treatment (50 µM) of 786-O and ACHN cells led to a significant decrease in MMP1 protein (Figure 4A) and mRNA expression (Figure 4B). As shown in Figure 4C, molecular docking analysis revealed that the binding energy between EA and MMP1 was significantly lower than -7.3 kJ/mol, indicating that EA has sufficient docking activity to directly interact with MMP1 proteins (Figure 4C). To determine the functional role of MMP1 in the mechanism underlying the effects of EA on RCC cells, we investigated these effects in the presence of overexpressed recombinant human MMP1 (Rh-MMP1). We found that EA treatment of 786-O and ACHN cells significantly decreased the migration and invasion of both lines of RCC cells (Figure 4D). Cells treated with 100 ng/mL Rh-MMP1 alone exhibited greater cell migration and invasion (Figure 4D). Combination treatment of RCC cells with EA and Rh-MMP1 reversed the effect seen with Rh-MMP1 alone, resulting in reduced cell migration and invasion (Figure 4D). These results show that EA inhibits RCC cell migration and invasion by targeting MMP1 expression.

### EA inhibit RCC cell migration and invasion through regulation of RUNX2 expression

**Figure 5:**
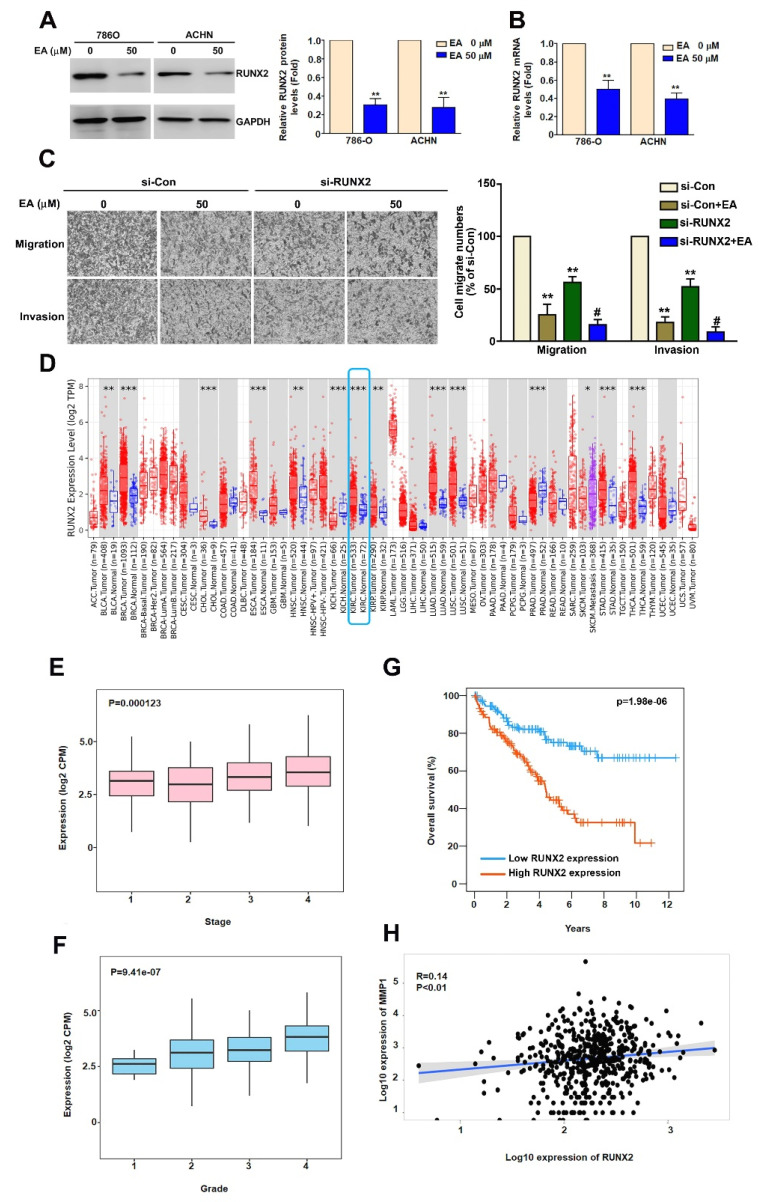
EA inhibits RUNX2 expression, which is associated with the migration and invasion of human RCC cells, and highlights the clinical significance of RUNX2. (A, B) Effect of EA treatment (50 µM) on RUNX2 mRNA and protein expression in RCC cell lines. (C) The inhibitory of migration and invasion in siRNA-RUNX2 (si-RUNX2) combined with EA in 786-O cells. ** p < 0.01 compared to untreated cells; # p < 0.05 compared to EA-treated cells. (D) Comparison of RUNX2 expression levels between normal and renal cell carcinoma tissues using Timer2.0 data. Comparison of (E) tumor stage, (F) tumor grade, and (G) 12-year overall survival rates between high and low RUNX2 gene expression levels in RCC tumor tissues. (H) Correlation between MMP1 and RUNX2 gene expression levels in RCC tumor tissues.

Studies have shown that RUNX2 is involved in tumor cell migration and invasion 23 and that RUNX2 directly targets the MMP1 promoter in TNBC cells 24. To determine whether EA inhibits RUNX2 expression in RCC cells, we used western blot and RT-qPCR analysis. We found that EA treatment significantly decreased RUNX2 protein and mRNA expression (Figure 5A, 5B). To clarify the role of RUNX2 in EA-treated RCC cells, we found that inhibition of RUNX2 using si-RUNX2 significantly reduced the migration and invasion abilities of EA-treated 786-O cells (Figure 5C). Further analysis of data in the TIMER2.0 database showed higher levels of RUNX2 expression in RCC tissues than in normal kidney tissues (Figure 5D). We also found that RUNX2 expression levels correlated with tumor stage (P = 0.000123) (Figure 5E), tumor grade (P = 9.41e-07) (Figure 5F), and overall survival (P = 1.98e-06) (Figure 5G). The results of Spearman correlation analysis indicate that RUNX2 expression positively correlates with MMP1 expression in human RCC tissues (R = 0.14; P = 0.01) (Figure 5H). These results show that EA inhibited RCC cell migration and invasion by downregulating RUNX2 targeting MMP1 expression, and clinical evidence suggests that the level of RUNX2 expression may be prognostic factor for RCC.

## Discussion

The results of this study show that EA treatment of RCC cells inhibits their invasion and migration capabilities, likely by mediating MMP1 expression levels. Our analysis of clinical data in the TCGA database shows that higher MMP1 and RUNX2 levels in tumor tissues are associated with higher tumor grade and stage and poorer overall outcomes of RCC patients. We found that EA, a naturally occurring compound, can retard metastatic behaviors in human RCCs by suppressing the expression of RUNX2, which targets MMP1 expression.

MMPs, when activated by growth factors and inflammatory cytokines, exert proteolytic activity that contributes to ECM degradation and remodeling 25, 26. Reorientation of collagen fibers in the ECM is associated with increased cancer invasion and even can predict worse survival in cancer patients 27. MMP1 (interstitial collagenase-1) is upregulated in many types of metastatic cancers, and an inverse relationship has been observed between MMP1 transcription levels and clinical outcomes 28, 29. Previous evidence and the results of the MMP1 is linked to RCC risk at the genetic level. One study indicated an association between an *MMP1* genetic variant and predisposition to kidney cancers 30. However, the correlation between polymorphisms in the MMP1 promoter region and risk of developing RCCs yielded conflicting results 31, 32. In human clear cell RCCs, higher grades of tumors were found to have lower collagen fiber content and higher collagenolytic activity of MMP1 33. One of the main results of our study shows that EA significantly decreased MMP1 expression in RCC cells; EA treatment of these cells also affected the expression of MMP2 and MMP9. The anti-invasive effects of EA on different malignant tumor types, exerted by mediating MMP expression, have been studied formerly. Colon carcinogenesis induced by 1,2-dimethyl hydrazine (DMH) was associated with augmented expression of MMP2 and MMP9, and EA co-treatment with DMH significantly reduced the protein expression of MMP2 and MMP9 34. In cultured human gastric cancer cells, EA treatment hindered the acidic microenvironment-induced upregulation of MMP7 and MMP9 mRNAs as well as tumor migration and invasion 35. The same study also reported that cyclooxygenase activity and the degree of EMT, both of which were increased under acidic conditions and correlated with tumor invasiveness, were inhibited by EA 35. Patients with late-stage RCCs have a low chance of survival despite standard anticancer therapies. Clinical trials of MMP1 inhibitors and drugs targeting other types of MMPs show that these are promising agents against metastatic cancers 36, 37. EA decreases the expression of MMP1 in RCC tumors, thereby inhibiting tumor invasion. Therefore, EA can be regarded as a potentially effective compound for treating metastatic RCCs; subsequent clinical studies are needed to evaluate the safety and efficacy of EA.

RUNX2 is a nuclear transcription factor involved in regulating osteoblast differentiation and chondrocyte maturation 38. Several studies have implicated RUNX2 in the progression of various malignant tumors. For example, high RUNX2 expression plays a significant oncogenic role in hepatocellular carcinoma 39, renal cell carcinoma 40, highly invasive breast cancer 41, and glioma 42. Guo *et al.* suggested that RUNX2 promotes gastric cancer tumorigenesis through YAP1 23. Additionally, studies on triple-negative breast cancer have confirmed that RUNX2 increases TGF-β-mediated regulation of CD44^+^/CD24^-^ breast cancer stem cells, leading to increased cancer stemness, EMT, and apoptosis resistance, as well as conferring resistance to epirubicin 43. Both *in vitro* and *in vivo* experiments have demonstrated that RUNX2 directly regulates MMP1 transcriptional activity, promoting TNBC tumorigenesis and increasing chemoresistance 24. In pancreatic cancer, RUNX2 has been shown to regulate the transcriptional activity of the extracellular matrix proteins SPARC and MMP1, thereby influencing the tumor microenvironment 44. In chondrosarcoma, IL-1β has been found to regulate p38, which in turn promotes RUNX2-mediated MMP-13 transcription and translation, playing a crucial role in tumor progression 45. Based on these findings, our study confirms that EA inhibits MMP1 expression by downregulating RUNX2, thereby suppressing the migration and invasion abilities of renal cancer cells. This study had a few limitations. We will investigate whether RUNX2 directly regulates MMP1 transcriptional activity and whether the *in vivo* metastasis mouse assay supports the anti-metastatic effect of EA observed *in vitro* remain to be further suggested in future studies.

Overall, our study demonstrated that the inhibitory effect of EA on renal cancer cell migration and invasion is mediated through the inhibition of the RUNX2/MMP1 axis. These findings provide novel and important reference data for elucidating the molecular mechanism underlying the inhibitory effect of EA on renal cancer cell migration and invasion, while also contributing to the development of clinical therapeutic strategies and new therapeutic targets for human RCC.

## Figures and Tables

**Figure 6:**
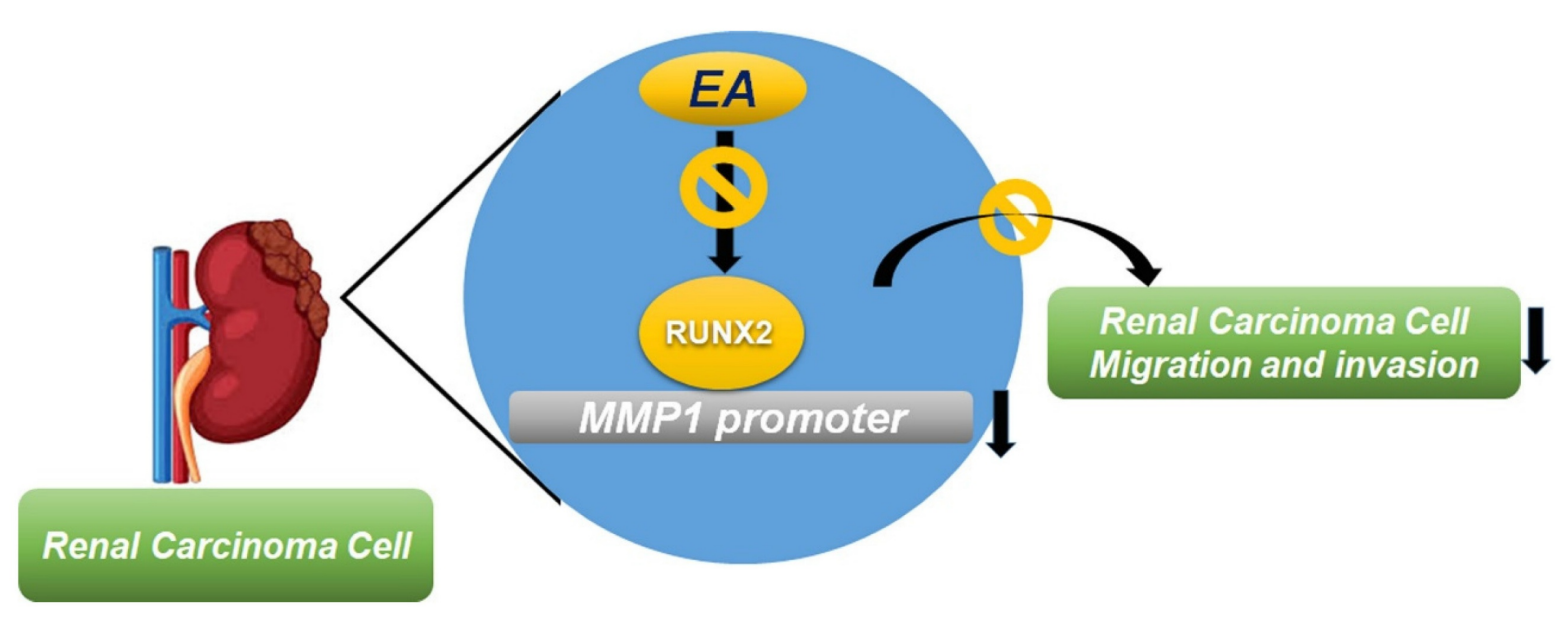
Summary of EA effects on RUNX2 regulation of MMP1 activity, which further suppresses the migration and invasion of RCC cells.
